# Rapid increase in neuroinvasive West Nile virus infections in humans, Italy, July 2022

**DOI:** 10.2807/1560-7917.ES.2022.27.36.2200653

**Published:** 2022-09-08

**Authors:** Flavia Riccardo, Antonino Bella, Federica Monaco, Federica Ferraro, Daniele Petrone, Alberto Mateo-Urdiales, Xanthi D Andrianou, Martina Del Manso, Giulietta Venturi, Claudia Fortuna, Marco Di Luca, Francesco Severini, Maria Grazia Caporali, Daniela Morelli, Federica Iapaolo, Ilaria Pati, Letizia Lombardini, Tamas Bakonyi, Olivia Alexandra, Patrizio Pezzotti, Maria Gabriella Perrotta, Francesco Maraglino, Giovanni Rezza, Anna Teresa Palamara, Italian Arbovirus Surveillance network, Italian Arbovirus Surveillance network, Dalia Palmieri, Manuela Di Giacomo, Michele La Bianca, Anna Domenica Mignuoli, Annunziata De Gaetano, Angelo D’Argenzio, Claudio Gualanduzzi, Matteo Giulio, Giovanna Mattei, Micaela Castelli, Francesco Vairo, Camilla Sticchi, Roberto Moschi, Danilo Cereda, Giulio Diurno, Lucia Crottogini, Daniel Fiacchini, Fabio Filippetti, Michele Colitti, Manuela Mariano, Silvia Spertini, Maria Grazia Zuccali, Chiara Pasqualini, Daniela Lombardi, Rosanna Prato, Maria Antonietta Palmas, Mario Palermo, Emanuela Balocchini, Francesco Angiò, Daniela Senatore, Simona Foresi, Mauro Ruffier, Francesca Zanella, Debora Ballarin, Francesca Russo, Maria Cristina Rota, Aurea Oradini Alacreu, Flavio Mellace, Stefania Giannitelli, Raffaele Donadio, Simonetta Pupella, Luciano Toma, Giovanni Savini, Maria Goffredo, Michela Quaglia, Annamaria Conte, Paolo Calistri, Daria Di Sabatino, Guido di Donato, Maria Teresa Scicluna, Giuseppe Manna, Davide Lelli, Mattia Calzolari, Francesco De Filippo, Roberto Iannone, Maurizio Viscardi, Loretta Masoero, Cristina Casalone, Nicola Cavaliere, Iolanda Padalino, Giantonella Puggioni, Giuseppa Purpari, Silva Costarelli, Monica Giammarioli, Stefano Gavaudan, Calogero Terregino, Fabrizio Montarsi, Gioia Capelli, Luisa Barzon

**Affiliations:** 1 Istituto Superiore di Sanità, Rome, Italy; 2 Istituto Zooprofilattico Sperimentale dell’Abruzzo e del Molise, Teramo, Italy; 3 Italian Ministry of Health, Rome, Italy; 4 European Centre for Disease Prevention and Control (ECDC), Stockholm, Sweden; 5 The members of the Italian Arbovirus Surveillance network are listed under Collaborators

**Keywords:** West Nile virus, Italy, One Health, surveillance, Italy, vector-borne infections, viral infections, outbreaks, public health policy, epidemiology

## Abstract

As in 2018, when a large West Nile virus (WNV) epidemic occurred, the 2022 vector season in Italy was marked by an early onset of WNV circulation in mosquitoes and birds. Human infections were limited until early July, when we observed a rapid increase in the number of cases. We describe the epidemiology of human infections and animal and vector surveillance for WNV and compare the more consolidated data of June and July 2022 with the same period in 2018.

West Nile virus (WNV) is endemic in Italy. Its circulation is currently monitored with a One Health approach that integrates human, animal and entomological surveillance aiming at timely detection of seasonal viral circulation. Italy adopted the European Union (EU) human case definition for WNV [1] which defines four laboratory criteria for case confirmation: isolation of WNV from blood or cerebrospinal fluid (CSF), detection of WNV nucleic acid in blood or CSF, WNV-specific antibody response (IgM) in CSF, high titre of WNV IgM AND detection of WNV IgG AND confirmation by neutralisation. In addition, Italy also recognizes detection of WNV nucleic acid in urine samples as a confirmatory WNV test [2]. A confirmed WNV human infection is therefore defined as any person meeting at least one of the listed laboratory criteria. Among all human cases of WNV infection, those presenting with neuroinvasive disease are further classified as cases West Nile neuro-invasive disease (WNND). The detection of WNV in any host or vector is confirmed by regional reference laboratories coordinated by the Ministry of Health, the Istituto Superiore di Sanità (epidemiology and national reference laboratory (NRL), human) and the Istituto Zooprofilattico of Abruzzo and Molise (epidemiology and NRL, animal/entomology).

During each transmission season, the first confirmed infections in humans, animals or mosquitoes trigger safety measures for substances of human origin (SoHO) coordinated by the National Blood and Transplant Centres. We describe the ongoing 2022 WNV transmission season, marked by early circulation and an unprecedented number of reported human cases, and compare the human cases reported in June and July 2022 with those reported in the same period of the 2018, when a severe WNV epidemic was documented in Italy [3,4].

## Epidemiological summary

**Figure 1:**
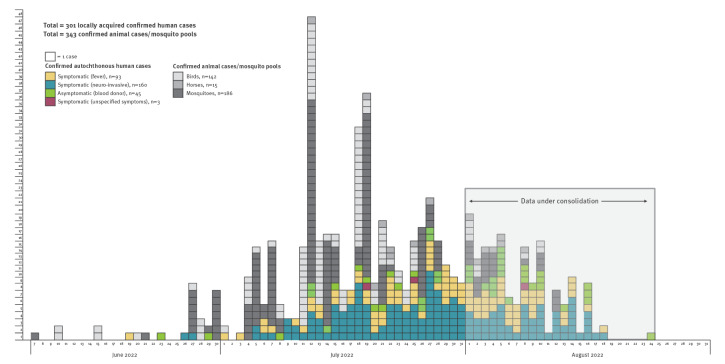
West Nile virus detection in mosquitoes, birds, horses and humans^a^, by date of detection or symptom onset, Italy, 1 June–24 August^b^ 2022

^a^ For all human cases, data are reported by date of onset except when not available (e.g. for non-symptomatic cases), in which case the date of diagnosis is presented.

^b^ August data are not consolidated.

The first detection of WNV circulation in 2022 occurred in early June [5] in mosquitoes and birds, with human cases detected from 19 June and rapidly increasing in July (Figure 1). Strains of WNV lineage 1 and 2 are co-circulating [5].

By 24 August 2022, WNV had been detected in 142 birds, 15 equids and 186 mosquito pools. Overall, 301 confirmed human infections, including 160 cases of WNND, had been notified to the Italian surveillance system (Figure 1). Detection in mosquitoes and vertebrate animals preceded that in humans except in 10 of the 41 Italian provinces affected so far. The data on infections reported or diagnosed in August 2022 are partial (as available by 24 August) and therefore not yet consolidated; they are likely to be incomplete due to reporting delays. For this reason, all comparisons with data reported in the 2018 epidemic year focus on the months of June and July. 

A total of 182 confirmed human infections had onset or, if unavailable, diagnoses up to 31 July including: 105 (57.7%) WNND cases of which 13 were fatal (case fatality rate (CFR): 12.4%), 54 West Nile fever (WNF) cases, 21 asymptomatic infections among blood donors and two cases with unspecified symptoms. The male:female ratio of those infections was ca 2:1 (117:65 among all confirmed human WNV infections and 66:39 among WNND cases). The median age of cases of WNV infection was 70 years (interquartile range (IQR): 56–78), 74 years in cases with WNND (IQR: 67–81) and 87 years in people who died with a WNV-related infection (IQR: 79–87).

During the 2018 epidemic year, Italy reported 162 human confirmed cases with onset or diagnosis up to 31 July including: 52 cases of WNND (32.1%) and 13 deaths (CFR: 25%), 89 cases of WNF and 21 asymptomatic infections.

## Trend and seasonality of human cases

**Figure 2:**
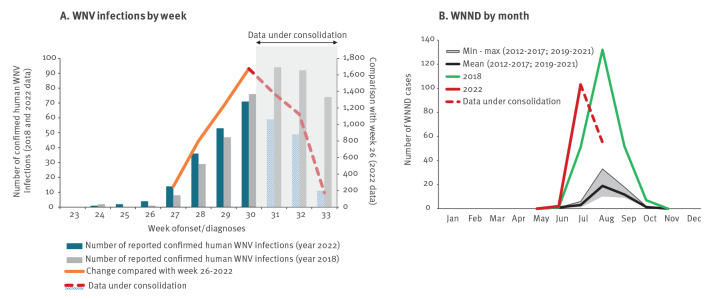
Confirmed human cases of West Nile virus infection notified in weeks 23–33 of 2022, (n = 300^a^) and of 2018 (n = 423) and confirmed cases of WNND, 2012–24 August 2022, Italy

WNND: West Nile neuro-invasive disease; WNV: West Nile virus.

^a^ One of the 301 WNV cases occurred on 24 August, i.e. after the period until week 33 shown in this graph.

Seven confirmed human WNV infections were notified with onset in June 2022 and up to the first week of July (w26: 27 June–3 July 2022). Compared with w26, we observed a 2.5-fold, 8-fold, 12-fold and 16-fold increase in the following 4 weeks. The trend of WNND cases in July exceeded the 2018 epidemic year (Figure 2).

Data updated 24 August (August 2022 data under consolidation). Dates refer to week or month of symptom onset or, if unavailable, diagnosis.

## Geographical distribution of human cases

**Figure 3:**
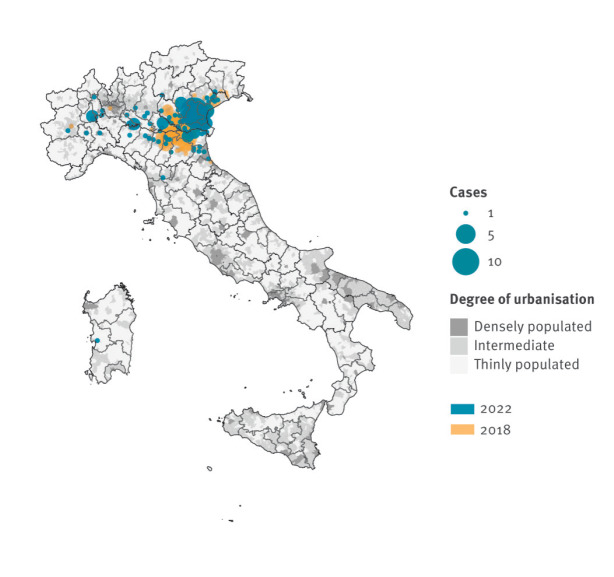
Geographical distribution of West Nile virus human infections in regions with at least one human case with onset or diagnosis up to 31 July, by affected municipality and degree of urbanisation, Italy, 2018 vs 2022

By 24 August 2022, 41 of the 107 Italian provinces had detected WNV circulation. Human cases with onset or diagnosis up to 31 July were reported in 25 provinces (128 municipalities) of the regions Emilia-Romagna, Friuli-Venezia Giulia, Lombardia, Piemonte, Sardegna, Toscana and Veneto. The highest human case counts were in Veneto (133 cases; 73%), mainly in the Padova province. In the same period of 2018, 24 provinces had detected WNV circulation, including 15 provinces (106 municipalities) with human cases. As shown in Figure 3, we observed in June and July 2022 a more westward and southward spread of human cases than in 2018, with more affected municipalities (mostly with an intermediate or low degree of urbanisation) (Table). 

Data updated 24 August 2022.

**Table:** **Table.** Number of municipalities with at least one human West Nile virus infection with onset or diagnosis up to 31 July, by degree of urbanisation, Italy, 2018 vs 2022

Degree of Urbanisation^a^	Number (%) of affected municipalities			
	2018		2022	
	n	%	n	%
Densely populated	11	10.4	12	9.4
Intermediate	62	58.5	62	48.4
Thinly populated	33	31.1	54	42.2
**Total**	**106**	**100**	**128**	**100**

Data updated 24 August 2022.

^a^ The degree of urbanisation per municipality used in the analysis is based on the EUROSTAT definition that accounts for geographical contiguity and population density [17,18].

## Discussion

Italy records on average 60 locally acquired human cases every year, mostly in the north of the country. In 2018, as in other European countries [6], Italy experienced an exceptionally early and intense WNV transmission [3] with over 600 confirmed human infections [4]. In 2022, until 24 August, most cases (76%) and deaths (71%) associated with WNV infections in the EU and European Economic Area (EEA) were reported by Italy [7], where the WNV transmission season started as early as in 2018 [5]. We observed an increase in the number of human WNND cases in July that exceeded what was observed in the same period of 2018.

Co-circulation of WNV lineages 1 and 2 was detected [5], while only lineage 2 circulation was documented in 2018 [3]. However, based on available data, there is no definite evidence of lineage-associated increased disease severity in humans. Considering this, and the fact that we did not observe an unusual demographic distribution of human cases in 2022, the higher proportion of notified WNND cases suggests increased under-detection or under-reporting of WNV infections. We hypothesise that the concurrent increased transmission of severe acute respiratory syndrome coronavirus 2 (SARS-CoV-2) may have made WNF ascertainment less likely, especially in the case of mild and self-limiting disease.

As in 2018, Italy has experienced climate anomalies in 2022. In 2018 higher-than-average temperatures in the month of April, preceded and followed by heavy rains across the country [8] might have led to an early and rapid increase in the mosquito population density and possibly an early amplification of viral transmission in the enzootic cycle [9]. Conversely, in 2022, climate anomalies occurred in May and June with higher-than-average temperatures in the context of persistent lower-than-average rainfall [10]. This has led to severe droughts in the Po valley [11] where WNV primarily circulates, followed by severe thunderstorms and floods from the beginning of July [12]. We hypothesise that the drought, possibly associated with regular irrigation in horticulture, might have limited the number of favourable habitats for mosquito breeding and bird nesting. This might have favoured an initial geographical concentration of vectors and hosts, viral amplification and, subsequently, the spread of WNV circulation once water became more abundant.

Following the 2018 outbreak, Italy performed an after-action review of its response [13] and issued an updated 5-year One Health National Arbovirus Disease Plan [2] which enhanced One Health surveillance and early warning, communication, SoHO safety and vector control across the country. This plan includes exceptional vector control measures with adulticides in the case of clusters of two or more spatio-temporarily linked human cases of WNND in intermediate or highly urbanised contexts [2].

In 2022, due to the increased transmission, all the planned measures were implemented, including exceptional vector control in heavily affected municipalities [14] and enhanced risk communication. Even through no transfusion-related cases have been detected, human cases triggered blood safety measures in 10 provinces, suggesting the need to assess and further improve early warning capacity from animal/vector surveillance.

## Conclusion

Italy, in contrast to other EU/EEA countries, is experiencing increased WNV transmission that exceeds 2018. Climate anomalies were also detected in other parts of the EU/EEA [15], and WNV circulation is currently increasing also in other European countries [16], in the context of mobility during summer that has returned to pre-pandemic levels. It is therefore important to raise public awareness to prevent exposure by adopting risk-mitigating behaviours. Raising awareness among clinicians can also encourage considering WNV among differential diagnoses in people with fever and/or neuroinvasive disease living in or returning from endemic areas.
